# Passive Samplers, a Powerful Tool to Detect Viruses and Bacteria in Marine Coastal Areas

**DOI:** 10.3389/fmicb.2021.631174

**Published:** 2021-02-23

**Authors:** Françoise Vincent-Hubert, Candice Wacrenier, Benjamin Morga, Solen Lozach, Emmanuelle Quenot, Mickaël Mège, Cyrielle Lecadet, Michèle Gourmelon, Dominique Hervio-Heath, Françoise S. Le Guyader

**Affiliations:** ^1^Ifremer, Laboratoire de Microbiologie, LSEM/SG2M, Nantes, France; ^2^Ifremer, Laboratoire de Génétique et Pathologie des Mollusques, LGPMM/SG2M, La Tremblade, France

**Keywords:** norovirus, Ostreid herpes virus 1 μvar, *Vibrio* spp., microbial source tracking, sea, passive sampler, oyster (*Crassostrea gigas*)

## Abstract

The detection of viruses and bacteria which can pose a threat either to shellfish health or shellfish consumers remains difficult. The current detection methods rely on point sampling of water, a method that gives a snapshot of the microorganisms present at the time of sampling. In order to obtain better representativeness of the presence of these microorganisms over time, we have developed passive sampling using the adsorption capacities of polymer membranes. Our objectives here were to assess the feasibility of this methodology for field detection. Different types of membrane were deployed in coastal waters over 2 years and the microorganisms tested using qPCR were: human norovirus (NoV) genogroups (G)I and II, sapovirus, *Vibrio* spp. and the species *Vibrio alginolyticus*, *V. cholerae*, *V. vulnificus*, and *V. parahaemolyticus*, OsHV-1 virus, and bacterial markers of fecal contamination. NoV GII, *Vibrio* spp., and the AllBac general *Bacteroidales* marker were quantified on the three types of membrane. NoV GII and OsHV-1 viruses followed a seasonal distribution. All membranes were favorable for NoV GII detection, while Zetapor was more adapted for OsHV-1 detection. Nylon was more adapted for detection of *Vibrio* spp. and the AllBac marker. The quantities of NoV GII, AllBac, and *Vibrio* spp. recovered on membranes increased with the duration of exposure. This first application of passive sampling in seawater is particularly promising in terms of an early warning system for the prevention of contamination in oyster farming areas and to improve our knowledge on the timing and frequency of disease occurence.

## Introduction

In a context of global change, monitoring microbial diversity in the coastal marine environment has become essential in terms of ecosystem health and impact. The presence of pathogens in coastal waters, linked to anthropogenic activities or naturally occurring in water, can pose a threat to shellfish health or shellfish consumers. Also, the quality of water in oyster farming areas can be affected by fecal pollution from urban and agricultural sources (Rince et al., 2018). In this context of water quality degradation, the protection of oyster production areas requires the detection of viruses and bacteria in seawater, as it is important to detect microorganisms in the water before they reach shellfish beds. Furthermore, effective resource management and remediation requires contamination sources to be identified. Microbial source tracking (MST), using *Bacteroidales* 16S rRNA gene markers, can identify the origins of fecal pollution in environmental waters or shellfish. However, these bacterial MST markers are weakly detected in shellfish and surrounding waters (Mauffret et al., 2013; Mieszkin et al., 2013). Current detection methods rely on point sampling of water and concentration of microorganisms to allow their subsequent detection with molecular techniques (Ikner et al., 2012). However, point sampling only gives a snapshot of the microorganisms present at the time of sampling, which may lead to underestimation of the risk of shellfish contamination especially for pathogens shed sporadically in the environment. Therefore, the development of *in situ* analysis tools, such as passive samplers, is of great importance to improve the detection of bivalve or human pathogens.

Passive samplers, largely used for the monitoring of dissolved chemical contaminants, are directly deployed in the field for several weeks to enable entrapment of the chemical contaminants (Vrana et al., 2005; Booij et al., 2016). The advantages of this approach are that it avoids handling and concentration of large volumes of water, it performs continuous and direct extraction of chemical contaminants from the water column, and provides a time-integrated sample of contamination, making it possible to integrate peaks of contamination as well as lower levels. The main advantage of integrative sampling is undoubtedly that it gives a level that is more representative of the real contamination than point sampling. Concerning microorganisms, very little use has been made of passive sampling; examples include the detection of poliovirus in wastewater or of norovirus in continental waters with gauze as the adsorption membrane (Fattal and Katzenelson, 1976; Tian et al., 2017). We have developed passive samplers constituted of membranes with capacity to adsorb viruses and bacteria (Vincent-Hubert et al., 2017). Following the adsorption phase, the detection of nucleic acids of different microorganisms is performed by PCR. For all membranes tested, except gauze, the quantity of nucleic acids of human norovirus (NoV) and Ostreid herpesvirus 1 (OsHV-1) adsorbed increased with the duration of exposure. These findings highlight the great potential of polymer membranes as passive samplers and possible future applications for field sampling.

As often reported, shellfish harvesting and bathing areas can be affected by fecal pollution from catchments areas including humans, livestock, pets, and wildlife (Le Mennec et al., 2017; Leight et al., 2018; Rince et al., 2018). General *Bacteroidales* and host-associated bacterial qPCR markers have been developed to identify fecal pollution and to distinguish fecal sources, i.e., human, bovine, porcine, avian, etc., respectively (Layton et al., 2006; Jarde et al., 2018). Among the human enteric viruses shed into the environment, NoV is currently the most frequent human pathogen detected in oysters and the most frequently implicated in gastroenteritis outbreaks linked to oyster consumption in Europe (EFSA, 2016). NoV-contaminated oysters have been detected worldwide following the malfunction of wastewater treatment plants during heavy rainfall or extreme weather events and subsequent contamination of marine waters. Sapovirus, another human enteric virus, is frequently detected in wastewater and has been associated with outbreaks of shellfish-associated illness (Nakagawa-Okamoto et al., 2009; Sima et al., 2011).

Pathogens naturally present in coastal waters, such as OsHV-1 and *Vibrio* species, can also impact shellfishery. The OsHV-1 virus is associated with mass mortality events of Pacific oysters, representing a major threat for oyster production. A new genotype of OsHV-1 called μVar has been reported in Europe as the main causative agent of mass mortality events affecting *Crassostrea gigas* (Segarra et al., 2010). *Vibrio* bacteria have been described from marine, brackish, and freshwater environments (Huq et al., 1983; Kirschner et al., 2008) and include many symbiotic and pathogenic species and strains. *Vibrio parahaemolyticus*, *V. vulnificus*, and *V. cholerae* are the three major human pathogenic species. However, more recently *V. alginolyticus* has been recognized as an emerging pathogen, with incidences of human infection rising significantly during summer months (Baker-Austin et al., 2016). These bacteria primarily cause gastroenteritis but are also known to cause wound and ear infections and primary septicemia (*V. vulnificus*). Actually, the detection of all these microorganisms is limited to oysters, whereas their early detection in water would mitigate their impact on oyster farming areas.

Our objectives here were to assess the feasibility of this new passive sampling methodology for field detection of microorganisms, to determine whether a particular type of membrane was more adapted to the field or to a microorganism, and to investigate whether the amount of nucleic acids adsorbed increased with exposure time. For this, three types of membrane, Zetapor, nylon, and low-density polyethylene (LDPE), were deployed for either 48 h or 15 days on a marine site. Two field samplings that followed the seasonal distribution of microorganisms were performed over 2 years. Microorganisms detected were NoV and sapovirus, OsHV-1, *Vibrio* spp. and *V. cholerae*, *V. vulnificus*, *V. parahaemolyticus*, and *V. alginolyticus*, and general fecal contamination and human-associated bacterial markers. We report here the detection and quantification of these microorganisms in a marine environment.

## Materials and Methods

### Preparation of Passive Samplers

Three different membranes, LDPE, Zetapor, and nylon, were used as passive samplers based on data obtained in a previous study (Vincent-Hubert et al., 2017). Zetapor filter (0.45 μm), an electropositive, charge-modified diatomaceous earth/cellulose filter, was purchased from 3M (Cergy-Pontoise, France), LDPE (thickness of 80 μm) from Manutan (France), and nylon nets (thickness of 100 μm) from Mougel (France). For LDPE and nylon membranes, pieces (4 cm × 25 cm) were cut from a roll and for Zetapor, six disks of 4.5 cm diameter were used. LDPE and nylon were directly attached on a device, and zetapor were put in a plastic mesh attached on the device (Supplementary Data Sheet 1). Membrane surfaces were 100 cm^2^ for LDPE and nylon and 96 cm2 for zetapor.

### Field Studies

**FIGURE 1:**
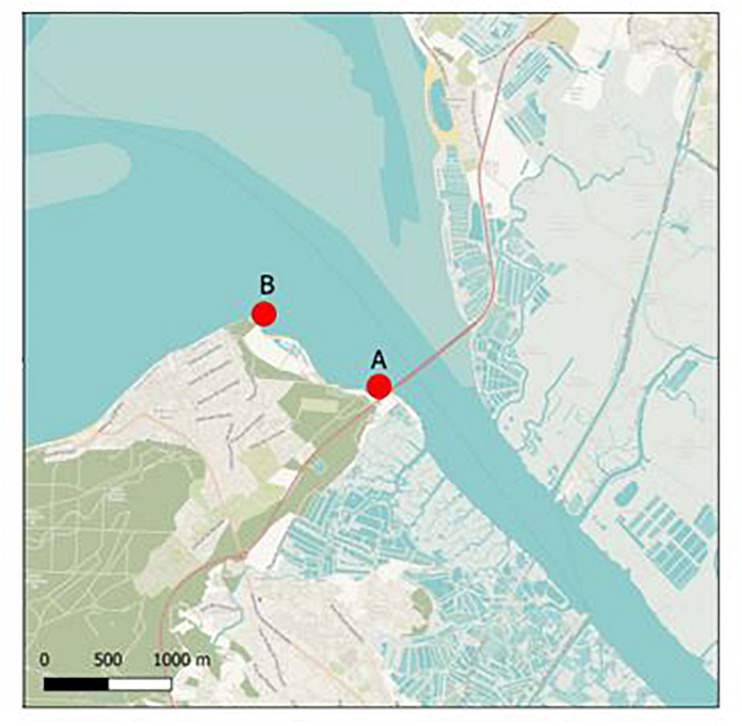
Sampling sites. Sampling sites (∙) in an oyster farming area. Site A is located 10 m downstream of a WWTP effluent outfall; site B is located 1,000 m downstream of the WWTP.

The two oyster farming sites selected for field studies were located in an estuary of the French Atlantic coast; site A was located approximately 10 m downstream of a waste water treatment plant (WWTP) effluent outfall and site B 1,000 m downstream of the WWTP (Figure 1). The estuary is mainly rural and has a population of 24,000 inhabitants. A first field study was performed for 8 months, from December 2016 to July 2017 (sites A and B) to test the passive sampling systems, and a second field study (site A only) was performed from October 2017 to October 2018. Membrane devices were fixed onto oyster tables on the foreshore and exposed to seawater for either 48 h or 15 days, which corresponds to an immersion time of about half that time due to the tidal cycle. After field deployment, membranes were collected and stored directly at −80°C until extraction of nucleic acids. Only four membrane devices were lost during field exposure, two in each period. We observed the presence of a slight biofilm on the 15 days exposed membranes only. Technological approach used for detection of virus and bacteria with passive sampling was detailed (Supplementary Data Sheet 2).

### Nucleic Acid Extraction

Membranes were rinsed in sterile water and nucleic acids were directly extracted from the membrane, the entire membrane was used for extraction. For the first field study, a NucliSENS extraction kit (bioMérieux, Lyon France) was used for NoV, *Vibrio* spp., and *Bacteroidales* markers, and a QiAamp tissue mini kit^®^ (QIAgen, France) was used for OsHV-1 as published previously (Vincent-Hubert et al., 2017). For the second field study, a single extraction procedure was used for all microorganisms using a NucliSENS extraction kit. Nucleic acids were eluted from the paramagnetic silica into 100 μl of elution buffer (bioMérieux, France), further purified using a Qiagen kit (RNA MinElute, Qiagen, France) to eliminate potential PCR inhibitors, and eluted with 120 μl of nuclease-free water (Qiagen, France).

### NoV and Sapovirus Detection and Quantification

Nucleic acid extracts were screened for human NoV (NoV GI and GII) by real-time RT-PCR (rRT-PCR) and sapovirus using previously published primers and probe (F. S. Le Guyader et al., 2009; Sano et al., 2011). rRT-PCR was performed on an MX3000 (Stratagene, Massy, France) using an UltraSense One-Step quantitative RT-PCR system (Invitrogen). All samples were analyzed in triplicate using 5 μl of undiluted or tenfold-diluted nucleic acid extracts. A negative amplification control (water) was included in each amplification series. Inhibitor removal was controlled by comparing the Ct values of pure and tenfold-diluted nucleic acid extracts, and by using a specific external control (ISO15216-1, 2017). Inhibitedsamples represented between 9.7and 16.4% of membranes exposed for 48 h and between 16.04 and 23.8% of those exposed for 15 days. The number of RNA copies present in positive samples was quantified using a standard curve based on an *in vitro* transcription plasmid containing nucleotides 4,191–5,863 of the Houston virus (GenBank EU310927) (F. S. Le Guyader et al., 2009). Only wells that yielded a Ct value of less than 39 were included in the quantitative analysis. Samples presenting a difference between Ct pure and Ct diluted (ΔCt) < 1 were quantified using mean Ct values. For samples presenting a ΔCt > 1, the Ct value used for quantification was the value obtained for the tenfold-diluted sample and then corrected using the slope of the standard curve. For a few samples (<7%), Ct values were between 39 and 40, thus under the limit of quantification; in those cases, quantification required substitution with LQ/2 value (LQ = 1.3 Log_10_) (EFSA, 2019).

### OsHV-1 DNA Detection and Quantification

OsHV-1 DNA detection and quantification PCR was performed in duplicate using an Mx3005P Thermocycler sequence detector (Agilent, France). Amplification reactions were performed in a total volume of 20 μl. Each well contained 5 μl of nucleic acid extracts from the membrane, 10 μl of Brilliant III Ultra-Fast SYBR^®^ Green PCR Master Mix (Agilent), 2 μl of each primer each at a final concentration of 550 nM, and 1 μl of distilled water (Webb et al., 2007). Real-time PCR cycling conditions were as follows: 3 min at 95°C followed by 40 cycles of amplification at 95°C for 5 s and 60°C for 20 s. Standard curve was prepared using dilutions of viral DNA suspension corresponding to a known amount of viral DNA copies extracted from purified virus particles. The standard curve included at least 5 concentrations of copies of OsHV-1 DNA (10^5^–10 cg/μl). The results were expressed as Log_10_ of virus OsHV-1 copy number of viral DNA/μl of DNA extract.

### *Vibrio* spp. Detection and Quantification

Total vibrios (*Vibrio* spp.) and *V. alginolyticus* were detected and quantified using a SYBR Green Real-Time PCR kit (Invitrogen; Fisher Scientific SAS, Illkirch Graffenstaden, France) using primers targeting the 16S rRNA region and the *dnaJ* gene, respectively (Thompson et al., 2004; Tall et al., 2012), *V. parahaemolyticus* using a TaqMan Real-Time PCR kit (Invitrogen) targeting the *toxR* gene, and *V. vulnificus* and *V. cholerae* using classical PCR as previously described (Chun et al., 1999; Hervio-Heath et al., 2002). Amplification was performed on an MXP3000 system (Stratagene, Massy, France). All samples were analyzed in triplicate using 2 μl of undiluted or tenfold-diluted nucleic acid extracts. A negative amplification control (water) was included in each amplification series. Quantification of vibrios was carried out using plasmid DNA standards (tenfold dilutions from 10^5^ to 10^2^ copies per PCR, with a limit of quantification (LQ) of ten target gene copies/reaction per PCR well) specific for each real-time PCR system.

### *Bacteroidales* Markers

General (AllBac) *Bacteroidales* and human-associated (HF183) *Bacteroidales* markers were detected and quantified using TaqMan Real-Time PCR (Platinum qPCR Supermix-UDG; Invitrogen; Fisher Scientific SAS, Illkirch Graffenstaden, France) and SYBR Green Real-Time PCR (Brilliant III Ultra-Fast SYBR QPCR Master Mix, Agilent Technologies, France) using primers targeting the 16S rRNA gene, respectively (Seurinck et al., 2005; Layton et al., 2006). A TaqMan exogenous internal positive control (IPC) reagent kit (Applied Biosystems, France) was added to the general *Bacteroidales* assays performed before the HF183 assays to distinguish true target negatives from PCR inhibition (Mauffret et al., 2012). All samples were analyzed in triplicate using 2 μl of undiluted or tenfold-diluted nucleic acid extracts. Negative controls (no template DNA) were performed in triplicate for each run. Linear DNA plasmids containing partial 16S rRNA gene sequence inserts were used as standards at tenfold dilutions ranging from 10^5^ to 10^2^ copies per PCR, with an LQ of ten and five target gene copies/reaction per PCR well for AllBac and HF183, respectively. Sample with values < LQ or with only one value from the triplicate assays > LQ were considered detected but not quantifiable (DNQ) and those with no amplification detected as not detected (ND). Amplification was performed on a CFX96 real-time system using Opticon Monitor version 3.1.32 and CFX manager version 1.1 software (Bio-Rad, France).

### Statistical Analysis

The frequency of positive membranes for each target microorganism (%) was calculated as follows: Number of positive membranes for each target microorganism/Number of samples analyzed for each target microorganism.

For site effect analysis, a generalized linear model (GLM), was performed to determine whether the frequency of positive membranes and the concentration were different. Statistical analyses were performed on the three microorganisms or bacterial marker which presented sufficient data: NoV GII, AllBac, and *Vibrio* spp. ANOVA or the Kruskal–Wallis test were performed to compare the concentration of gene or genome copies/membrane (gc/membrane) according to the membrane type or duration of exposure; the Tukey HSD test was used for pairwise comparisons. The frequency of detection of NoV GII and AllBac were analyzed with a GLM, with month and membrane as factors. NoV concentrations were compared with Student’s *t* test (autumn-winter compared with spring-summer). Three levels were considered significant: *p* < 0.05 (^∗^), *p* < 0.01 (^∗∗^), and *p* < 0.001 (^∗∗∗^). All statistical analysis and data plotting was performed with R Studio v 3.6.

## Results

### Detection of Microorganisms on Membranes

An overview of all the microorganisms detected on all membranes during the two field monitoring periods is presented in Table 1. Seven of the ten target microorganisms were detected by any type of membrane. The frequencies of positive membranes, for all types of membranes combined, varied according to the target microorganism: positive membranes were observed more frequently for *Vibrio* spp., the general *Bacteroidales* marker AllBac, NoV, and *V. alginolyticus*, illustrating the predominance of these microorganisms in the environment; membranes were less frequently positive for OsHV-1, sapovirus, and the human-associated *Bacteroidales* marker HF183. *V. parahaemolyticus*, *V. vulnificus*, and *V. cholerae* were never detected. If we consider the type of membrane, the frequencies of positive membranes were similar for *Vibrio* spp., NoV, and the general *Bacteroidales* marker AllBac, suggesting that the performance of the three types of membrane is equivalent. However, Zetapor was more frequently positive (*p* < 0.001) for OsHV-1 and *V. alginolyticus*, and LDPE and nylon membranes were more frequently positive for the human-associated *Bacteroidales* marker HF183, suggesting that the type of membrane can influence adsorption. Sapovirus was the least frequently detected microorganism, present only on nylon and Zetapor.

**TABLE 1:** Frequencies of positive membranes for each target microorganism percentages calculated for 48 h and 15 days of exposure.

	Period analyzed	All membranes	LDPE	Nylon	Zetapor
OsHV-1	Spring-summer *n* = 132	7.5	4.5	2.3	16
*Vibrio* spp.	All year *n* = 167	100	100	100	100
*V. alginolyticus*	Spring-summer *n* = 89	32.5	38.5	27	53.9
Sapovirus	All year* *n* = 84	4.7	0	6.9	7.4
NoV	All year *n* = 241	36	32.5	35	40.7
AllBac	All year *n* = 179	72	70	74.1	72.1
HF183	All year *n* = 134	13.5	17.8	13.9	7.14

*Sapovirus was analyzed only in 2017. AllBac, General Bacteroidales marker; HF183, human-associated Bacteroidales marker.

Concerning OsHV-1, in 2017, the virus was detected essentially on 48 h-exposed membranes from March to June, except in May, and in 2018 from April to August (Table 2). OsHV-1 was rarely detected on 15 day-exposed membranes, only in June and September 2018.

**TABLE 2:** Detection of OsHV-1 on membranes exposed for 48 h and 15 days.

		March	April	May	June	July	August	Sept	Oct
2017	48 h	+	+	–	+	NA	NA	NA	NA
	15 days	–	–	–	–	NA	NA	NA	NA
2018	48 h	–	+	+	+	+	+	–	–
	15 days	–	–	–	+	–	–	+	–

*+, detected; −, not detected; NA, not analyzed because no membrane was deployed.*

### Quantification of Microorganisms on Membranes and Occurrence

#### Site Effect Analysis for the 2016–2017 Monitoring Period

For the first monitoring period (2016–2017), membranes were collected from two different sites while only one site was selected for the second monitoring period (2017–2018). A binomial GLM was performed to determine whether the frequency of positive membrane and the concentration were different for each month for NoVGII, AllBac and HF183. No site effect was observed for NoVGII and AllBac, and a slight effect was observed for HF183 who was more frequently detected on site A, the site located 10 m downstream of a WWTP effluent outfall when compared to site B (*p* < 0,1).

The mean concentrations over the year were the same between the two sites for all microorganisms (Supplementary Data Sheet 3). No significant variation was observed concerning the exposure time (data not shown). As we did not observe any significant influence of the site on the quantifiable markers, all data were compiled in the same dataset for the 2016–2018 period for further statistical analysis.

#### Quantification of Microorganisms for the 2016–2018 Period

##### NoV GII

**FIGURE 2:**
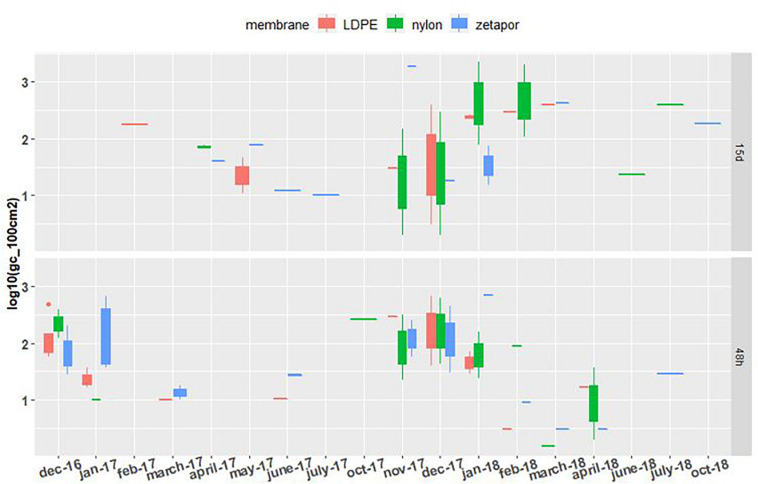
NoV GII concentrations on membranes exposed for 48 h and 15 days. Boxplots show the minimum, 25th percentile, median, 75th percentile, and maximum concentration per month. 48 h: membranes exposed for 48 h; 15 days: membranes exposed for 15 days. Limit of detection = 1.3 Log_10_. For the 2016–2017 period, no sampling was performed in February and May 2017 for the 48 h-exposed membranes, or in December, January, and June for the 15 day-exposed membranes. For the 2017–2018 period, sampling was performed every 2 weeks throughout the year.

NoV RNA was detected and quantified on the three types of membrane during the whole 2016–2018 period, with concentrations ranging from 1.3 to 3.6 Log_10_ genome copies/100 cm^2^ of membrane (gc/100 cm^2^) (Figure 2). For the autumn-winter period, the concentrations on all membranes were higher compared to the spring/summer period, when the virus was less detected and quantified even below the LQ (1.3 Log_10_ gc/100 cm^2^ of membrane). From May to October, on 48 h-exposed Zetapor membrane, the virus was detected on only one membrane in June 2017 and July 2018; on 15 day-exposed membranes (nylon and Zetapor), the virus was detected only once in May 2017 and June, July, and October 2018 (Figure 2). As expected, the presence of virus on a membrane seems to vary according to the season; NoV distribution was analyzed by a binomial GLM. The data obtained were converted to binary form and the relationship between the month/membrane type and presence/absence of NoV was assessed. The analysis showed that NoV was significantly more likely to be detected on membranes in the late autumn and early winter (November 2017, December 2016 and 2017, and January 2017 and 2018) (*p* < 0.001); no significant influence of membrane was observed. Moreover, a significantly higher concentration of NoV was observed on 48 h-exposed membranes during the autumn-winter period (2016–2018) compared with spring-summer, confirming the seasonality of NoV (*p* < 0.01) (Supplementary Data Sheet 4).

##### *Vibrio* spp.

**FIGURE 3:**
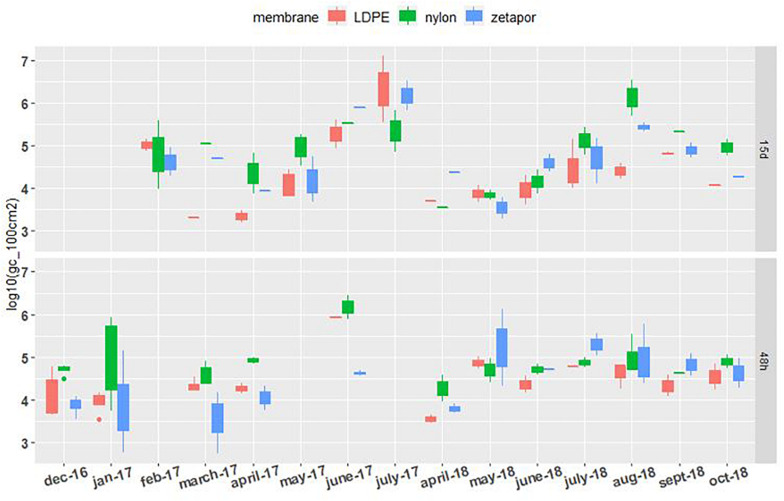
*Vibrio* spp. concentrations on membranes exposed for 48 h and 15 days. Boxplots show the minimum, 25th percentile, median, 75th percentile, and maximum concentration per month. 48 h: membranes exposed for 48 h; 15 days: membranes exposed for 15 days. For the 2016–2017 period, no sampling was performed in February and May for the 48 h-exposed membranes, or in December, January, and June for the 15 day-exposed membranes. For the 2017–2018 period, sampling was performed every 2 weeks from April to October.

Total vibrios (*Vibrio* spp.) were quantifiable on the nylon, LDPE, and Zetapor membranes during the two monitoring periods (2016–2017 and 2017–2018) (Figure 3). For 48 h-exposed membranes, concentrations of *Vibrio* spp. did not vary as much as those observed for membranes exposed for 15 days. A slight increase in concentration was seen between April and June of each year (2017 and 2018). In contrast, on membranes exposed for 15 days, *Vibrio* spp. concentrations varied a lot more, from 3.5 Log_10_ to 7.1 Log_10_ gc/100 cm^2^. A clear seasonal distribution of *Vibrio* spp. was observed in 2017 and 2018, showing a progressive increase from month to month from spring to late summer. The highest *Vibrio* spp. concentration was measured with LDPE (7.2 Log_10_ gc/100 cm^2^).

### General *Bacteroidales* Marker AllBac

**FIGURE 4:**
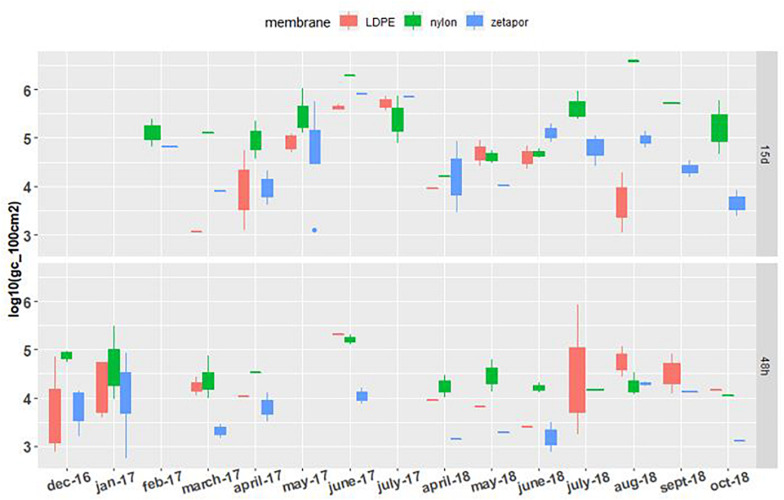
AllBac concentration on membranes exposed for 48 h and 15 days. Boxplots show the minimum, 25th percentile, median, 75th percentile, and maximum concentration per month. 48 h: membranes exposed for 48 h; 15 days: membranes exposed for 15 days. For the 2016–2017 period, no sampling was performed in February and May 2017 for the 48 h-exposed membranes, or in December, January, and June for the 15 day-exposed membranes. For the 2017–2018 period, sampling was performed every 2 weeks from April to October.

The general *Bacteroidales* marker AllBac was detected and quantifiable on LDPE, Zetapor, and nylon membranes during the entire monitoring period whatever the exposure time (Figure 4). For membranes exposed for 48 h, the concentration of AllBac was quite stable between 3.3 and 5.2 Log_10_ gc/100 cm^2^ with no marked seasonal variation. The highest concentrations of AllBac were measured on nylon membrane in June 2017 and on LDPE in July 2018, 5.2 and 5.9 Log_10_ gc/100 cm^2^, respectively. The lowest concentrations of these markers were always measured with Zetapor membrane. For membranes exposed for 15 days, the concentration was less stable than on 48 h-exposed membranes: an increase was clearly observed from March to July 2017 and again from April to July 2018 (Figure 4). The highest concentrations were measured with nylon membrane in June 2017 and August 2018, 6.3 and 6.6 Log_10_ gc/100 cm^2^, respectively. As AllBac was not detected every month with all membranes, we performed a binomial GLM who showed that AllBac was more frequently detected on nylon membrane (*p* < 0,01).

#### Analysis of the Influence of Membrane Performance and Exposure Time

##### NoV

**FIGURE 5:**
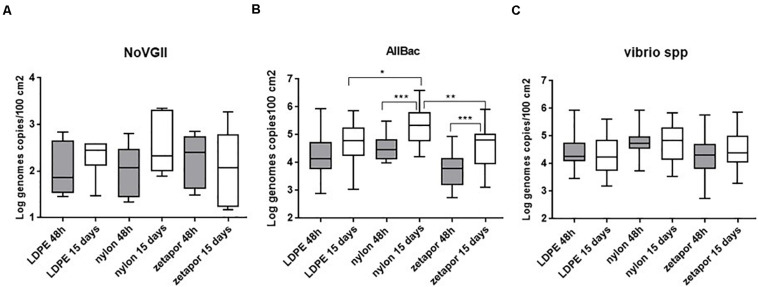
Comparison of NoV GII **(A)**, AllBac **(B)**, and *Vibrio* spp. **(C)** concentrations on membranes. Boxplots show the minimum, 25th percentile, median, 75th percentile, and maximum concentration per month. For NoV GII, concentrations are reported for the 2017–2018 dataset. For *Vibrio* spp. and AllBac, concentrations are reported for the 2016–18 dataset. + : Mean concentrations. *p < 0.05; **p < 0.01; ***p < 0.001. ANOVA was used for AllBac dataset.

We tested the effect of exposure time and membrane on the winter-spring period of the 2017–2018 dataset, which is a complete dataset since the implementation of field sampling was more effective than during the first year. No significant effect of these two factors influenced the concentration of the virus (Figure 5A). However, the frequencies of NoV-positive nylon membranes (48 h and 15 days) were always, but not significantly, higher than for the other membranes (Supplementary Data Sheet 5).

##### *Vibrio* spp. and AllBac

The membrane performance and the effect of exposure time were analyzed on the complete dataset (2016–2018) to determine its potential influence on the concentration. For total vibrios (*Vibrio* spp.), the concentrations measured on nylon membrane were higher but not significantly higher than those on Zetapor and LDPE only (Figure 5C). No significant effect of exposure time was observed (Figure 5C).

For AllBac, analysis of membrane performance and exposure time revealed that the concentration was significantly higher on nylon membrane than those on Zetapor and LDPE for 15 days exposure only, suggesting that nylon is more efficient for passive sampling than the two other types of membrane (*p* < 0.001) (Figure 5B). Concerning exposure time, a significant effect was observed for nylon compare with LDPE and zetapor, with 15 days’ exposure being more efficient for passive sampling of AllBac with nylon (*p* < 0.01 and *p* < 0.05).

## Discussion

The detection of viruses and bacteria in a marine environment requires high-performance methods capable of detecting low concentrations and, ideally, variations in these concentrations. The results presented in this article clearly illustrate the value of passive samplers, combined with molecular detection, for the direct detection of some microorganisms that are usually detected in shellfish but rarely in seawater. Passive sampling has rarely been applied for detection of viruses and bacteria, and the only field studies published used a cotton membrane, with the Moore swab method (Cooley et al., 2013; Tian et al., 2017). For NoV, we obtained a detection rate of 34% in seawater, which is relatively similar to results published for freshwater (26%) (Tian et al., 2017) and the concentration on the membrane is quite similar to those found in seawater with point sampling (La Rosa et al., 2009; Rusinol et al., 2015; Kaas et al., 2016), which shows the interest of this method. NoV GI was rarely detected on membranes, confirming our previous results showing a tenfold difference in seawater concentration between NoV GI and GII, while the opposite is observed in oysters (Maalouf et al., 2010). This highlights the interest of monitoring all the different pathogens in the water column as shellfish can selectively accumulate some of these pathogens (Le Guyader et al., 2012; Morozov et al., 2018).

The method developed here gives integrated sampling over time for the more frequently detected microorganisms, AllBac, NoV, and *Vibrio* spp., which is a characteristic of passive samplers in general (Taylor et al., 2019). These observations are consistent with our laboratory experiments in which we observed that the concentration of NoV on membranes increased with the duration of exposure (Vincent-Hubert et al., 2017). The integrative nature of microorganism sampling using membranes is also shown for an enveloped virus, OsHV-1, supposedly present at low concentrations in the marine environment. This finding is even more important as enveloped viruses are less environmentally persistent than their non-enveloped counterparts and therefore more difficult to detect in seawater.

Integrative sampling is particularly interesting for microorganisms present at low or variable concentrations in seawater, such as OsHV-1 and NoV. In these particular cases, passive sampling could improve the estimates made by point sampling or could substitute for composite samples. Composite samples have been suggested as an alternative to point sampling to improve the concentration estimates for enteric viruses as their concentration may vary during the same day, but this approach may be difficult to set up in the field (Gerba et al., 2017; Farkas et al., 2018). Indeed, the main interest of our system is that it detected NoV during the whole autumn-winter season, whereas we did not detect the virus with point sampling although the sampling site was located downstream of the WWTP effluent outfall (data not shown). In the same way, passive samplers allow the detection of OsHV-1 before mortality events, while this virus is hardly detected in seawater even though it is frequently detected in infected or asymptomatic oysters (Segarra et al., 2010; Evans et al., 2014). All these findings suggest that time-integrated sampling could facilitate the detection of low concentrations of viruses as well as emerging viruses as their presence and fate in water is hindered by the lack of proven detection methods (Wigginton et al., 2015).

Passive sampling allowed observation of the seasonality of microorganisms, as illustrated by the NoV and OsHV-1 viruses and, to a lesser extent, with bacteria. We found that NoV GII was dominant on membranes during the winter season, which correlates with other studies showing that this genogroup is the predominant cause of human gastroenteritis during the winter season (Tran et al., 2013). The presence of human NoV in coastal environments is due to discharge of WWTP effluent, and its distribution in the environment is dependent on the season and linked to the epidemiology (Kitajima et al., 2014; Atmar et al., 2018). OsHV-1 was detected on membranes, revealing its presence in seawater during the spring-summer season, the usual period of OsHV-1 infection in oysters. For bacteria, membranes allowed the direct detection of *Vibrio* spp. at various concentrations all year round and of *V. alginolyticus* during the spring-summer season. Detection of these bacteria on the 15 day-exposed membranes confirmed the seasonal dynamics of *Vibrio* spp. observed previously in European coastal waters using cultural methods (Oberbeckmann et al., 2012; Cantet et al., 2013). Recent data have shown that the incidence of *Vibrio*-associated illnesses is increasing worldwide and an unprecedented number of domestically acquired human infections are associated with swimming/bathing in coastal waters (Schets et al., 2011; Baker-Austin et al., 2013). The general *Bacteroidales* fecal marker AllBac was detected all year round, with a tendency to seasonality in spring-summer as revealed with the 15 day-exposed membranes. The frequent and high quantification of this marker on the membranes clearly shows the presence of bacteria of the order *Bacteroidales* in these marine waters.

Some microorganisms were poorly or not detected on membranes, such as *V. parahaemolyticus*, *V. vulnificus*, and *V. cholerae* indicating the limit of passive sampling for these potential human pathogens. Other microorganisms were poorly detected and non-quantifiable, such as the human-associated *Bacteroidales* marker HF183 at the site directly impacted by WWTP effluent and sapovirus. These low concentrations of microorganisms on the membranes are probably proportional to their concentration in the marine water column. Indeed, the human marker HF183 has been detected less frequently and most often at lower concentrations in marine water than in river water upstream in a coastal catchment area (Jarde et al., 2018). It can also be explained by the degradation of microorganisms by UV light. UV degradation could occur because the membranes are only submerged during high tide; such a drawback can be avoided in the future by selecting sites where membranes will be constantly immersed, thus possibly allowing more microorganisms to be recovered.

The detection of microorganisms on membranes is the result of attachment of the microorganisms onto the membranes and to the success of their molecular detection. Our data show that specificity of the membranes toward a microorganism is not evident for pathogens frequently detected (i.e., NoV, *Vibrio* spp.), while it seems determinant for OsHV-1 that is detected exclusively with Zetapor. However, for the more frequently detected microorganisms, nylon membrane seems to be more efficient if we consider the frequency of detection and the concentration of microorganism per membrane. The attachment of virus and bacteria is probably influenced by the presence of biofilm observed on the 15 days exposed membranes, as observed for example with norovirus on natural biofilm (Skraber et al., 2009). Moreover, with favorable condition of temperature *Vibrio* spp. might grow and formed a biofilm which could explain the increase observed on 15 days exposed membranes. Thus, short exposure time probably better reflects the concentration of these microorganisms in the environment.

The mechanisms by which microorganisms attach onto surfaces in seawater are not well understood. They are probably influenced by the physicochemical characteristics of the microorganism (i.e., isoelectric point, particle size), of the membrane (i.e., electric charge, hydrophobicity), and of seawater (pH, ionic strength) (Lobelle and Cunliffe, 2011; Amaral-Zettler et al., 2020; Yamada et al., 2020). Concerning their molecular detection, another advantage of using passive sampling is that the presence of PCR inhibitors, either biological molecules or particulate organic matters, seems to be limited, as exemplified by our data with an inhibition rate ranging from 9.7 to 23%.

Our results raise the question of the potential for viruses to attach to abiotic surfaces in seawater, while bacterial attachment to marine plastic debris has already been described (Amaral-Zettler et al., 2020). Our data show that bacterial colonization occurs as soon as 48 h of exposure, which is information additional to that of Harrison and collaborators who showed bacterial colonization after as little as 1 week on LDPE microplastics in coastal marine sediments (Harrison et al., 2014). LDPE, as other plastic debris in marine waters forming the so called “plastisphere,” has the potential to carry microbial agents (Amaral-Zettler et al., 2020; Turetken et al., 2020). Among the microorganisms found on plastic debris, *Vibrio* species are dominant, with some pathogenic *Vibrio* detected, while viruses have never been reported (Zettler et al., 2013; Kirstein et al., 2016). Our data illustrate the capacity of viruses to attach to polymer surfaces, making it possible to investigate the presence of viruses in the plastisphere. We previously demonstrated that viruses (NoV and OsHV-1) are adsorbed onto polymer surfaces and we depicted their adsorption rate on membranes and their stability, which can explain our current observations (Vincent-Hubert et al., 2017). Attachment of NoV onto membranes is probably facilitated by its interaction with bacteria and particulate matter [review in Amarasiri and Sano (2019)]. Indeed, specific interaction of NoV involving histo-blood group antigens (HBGAs) has been found in *Enterobacter cloacae* and in some bacteria of the human gut microbiome (Miura et al., 2013; Almand et al., 2017).

## Outlook

This first application of passive sampling is particularly promising in terms of early detection of viruses, of human or marine origin, to mitigate contamination in oyster farming areas and to improve our knowledge of the life cycle and diversity of viruses in seawater. The current results tend to demonstrate the relevance of using passive sampling for assessing the presence of *V. alginolyticus*, eventhough the method needs to be improved. This tool could also be used to monitor the emergence and presence of pathogens involved in mass mortality of marine life in the coastal marine environment (Vezzulli et al., 2010).

## Conclusion

We demonstrated that passive sampling coupled to molecular detection is a powerful new method for the detection of natural and anthropic viruses and bacteria in coastal environments. Our data show that viruses, NoV, sapovirus, and OsHV-1 can be detected on polymer surfaces immersed in seawater and confirm that bacteria are highly represented on LDPE. Nylon membrane seems to be more performant for the detection of viruses and bacteria, except for OsHV-1, for which Zetapor is preferred. Immersion of the membranes for 48 h to 15 days gives integrated sampling over time (Supplementary Data Sheet 6). However, short exposure time would probably better reflects the concentration of virus and bacteria in the environment by limiting the formation of biofilm, the degradation of viruses unstable in the environment such as OsHV-1 and the presence of PCR inhibitors. Depending on the purpose of the study, detection may be sufficient to determine the occurrence; however, quantification of microorganisms could be performed on a larger data set to provide additional information for monitoring study.

## Data Availability Statement

The raw data supporting the conclusions of this article will be made available by the authors, without undue reservation.

## Author Contributions

FV-H: supervised, statistical analysis, molecular analysis, and writing. CW, EQ, FL, MM, and CL: molecular analysis. DH-H, MG, SL, and BM: discussion. All authors contributed to the article and approved the submitted version.

## Conflict of Interest

The authors declare that the research was conducted in the absence of any commercial or financial relationships that could be construed as a potential conflict of interest.

## Supplementary Material

Supplementary Data Sheet 1 Device used for passive sampling. Zetapor (left), nylon and LDPE (right) membranes. zetapor dics were put in a plastic mesh bag attached on the crate while LDPE and nylon membranes were directly attached on the crate.

Supplementary Data Sheet 2 Technological approach used for detection of virus and bacteria with passive sampling.

Supplementary Data Sheet 3 Mean concentrations over the year of microorganisms at the two sites in 2016–2017.

Supplementary Data Sheet 4 Comparison of NoV GII concentrations on membranes in autumn-winter and spring-summer. The mean concentration of NoV GII ± SD on membrane was calculated for two periods, autumn-winter (A-W) and spring-summer (S-S) for all types of membrane combined (^∗^*p* < 0.05).

Supplementary Data Sheet 5 Frequencies of NoV GII-positive membrane in 2017–2018. Frequencies are expressed as percentages.

Supplementary Data Sheet 6 Recommendation for passive sampling according to microorganisms.
