# Supplementary figures and images for: Passive Samplers, a Powerful Tool to Detect Viruses and Bacteria in Marine Coastal Areas
Source: Front Microbiol. 2021 Feb 23;12:631174. doi: 10.3389/fmicb.2021.631174 (PMC7940377; doi:10.3389/fmicb.2021.631174)

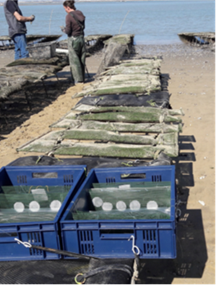
**
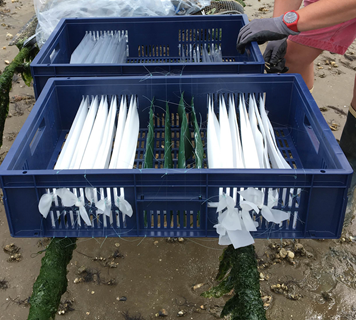
**

Supplement: Supplementary Data Sheet 1 — Device used for passive sampling. Zetapor (left), nylon and LDPE (right) membranes. zetapor dics were put in a plastic mesh bag attached on the crate while LDPE and nylon membranes were directly attached on the crate. [file Data_Sheet_1.DOCX]
