# Supplementary material for: Passive Samplers, a Powerful Tool to Detect Viruses and Bacteria in Marine Coastal Areas
Source: Front Microbiol. 2021 Feb 23;12:631174. doi: 10.3389/fmicb.2021.631174 (PMC7940377; doi:10.3389/fmicb.2021.631174)
Supplement: Supplementary Data Sheet 2 — Technological approach used for detection of virus and bacteria with passive sampling. [file Data_Sheet_2.DOCX]

|  | **Monitoring (1=2016-2017; 2=2017-2018)** | **Period analysed** | **sites** | **NA extraction methods** | **Target gene detection and Quantification** | **Theoritical limit of detection *** (gc/membrane) | **Reference** |
| --- | --- | --- | --- | --- | --- | --- | --- |
| **OsHV-1** | 1  2 | Spring-summer  Spring-summer | A, B  A | QiAamp tissue mini kit  NucliSENS | SYBR Green Real-Time PCR | 200 | Vincent-Hubert, F. *et al.,* 2017 |
| ***Vibrio* spp.** | 1  2 | Dec-July  April-October | A, B  A | NucliSENS  NucliSENS | SYBR Green Real-Time PCR | 200 | Thompson *et al.,* 2004 |
| ***V. alginolyticus*** | 1  2 | Spring-summer  Spring-summer | A, B  A | NucliSENS  NucliSENS | SYBR Green Real-Time PCR | 200 | Tall et al., 2013 |
| ***V. cholerae*** | 1  2 | Spring-summer  Spring-summer | A, B  A | NucliSENS  NucliSENS | SYBR Green Real-Time PCR | 200 | Chun et al., 1999 |
| ***V. vulnificus*** | 1  2 | Spring-summer  Spring-summer | A, B  A | NucliSENS  NucliSENS | SYBR Green Real-Time PCR | 200 | Tall et al, 2013 |
| ***V. parahaemolyticus*** | 1  2 | Spring-summer  Spring-summer | A, B  A | NucliSENS  NucliSENS | TaqMan Real-Time PCR | 200 | Hervio-Heath *et al*., 2002 |
| **Sapovirus** | 1  no | Dec-July | A, B | NucliSENS | TaqMan Real-Time PCR | 200 | Sano *et al.,* 2011 |
| **NoV** | 1  2 | Dec-July  October-October | A, B  A | NucliSENS  NucliSENS | TaqMan Real-Time PCR | 200 | Vincent-Hubert. *et al.,* 2017 |
| **AllBac** | 1  2 | Dec-July  April-October | A, B  A | NucliSENS  NucliSENS | TaqMan Real-Time PCR | 500 | Maufret *et al.,* 2012 |
| **HF183** | 1  2 | Spring-summer  Spring-summer | A, B  A | NucliSENS  NucliSENS | SYBR  Green Real-Time PCR | 250 | Maufret *et al.,* 2012 |

**Supplementary Data 2  : Technological approach used for the detection of virus and bacteria with passive sampling ****Theoritical limit of detection in pure sample*
