# Supplementary material for: Passive Samplers, a Powerful Tool to Detect Viruses and Bacteria in Marine Coastal Areas
Source: Front Microbiol. 2021 Feb 23;12:631174. doi: 10.3389/fmicb.2021.631174 (PMC7940377; doi:10.3389/fmicb.2021.631174)
Supplement: Supplementary Data Sheet 3 — Mean concentrations over the year of microorganisms at the two sites in 2016–2017. [file Data_Sheet_3.DOCX]

|  | **Frequency** | | **Mean concentration** | |
| --- | --- | --- | --- | --- |
|  | **Site A** | **Site B** | **Site A** | **Site B** |
| **NoV GII** | 40.5 | 37.8 | 2.1 ± 2 | 1.7 ± 1.6 |
| **AllBac** | 92 | 86 | 5.1 ± 5.0 | 5.2 ± 5.0 |
| **HF183** | 17.8* | 6.1* | DNQ | DNQ |
| ***Vibrio* spp.** | 100 | 100 | 5.2 ± 5.0 | 5.3 ± 5.6 |
