# Supplementary material for: Passive Samplers, a Powerful Tool to Detect Viruses and Bacteria in Marine Coastal Areas
Source: Front Microbiol. 2021 Feb 23;12:631174. doi: 10.3389/fmicb.2021.631174 (PMC7940377; doi:10.3389/fmicb.2021.631174)
Supplement: Supplementary Data Sheet 4 — Comparison of NoV GII concentrations on membranes in autumn-winter and spring-summer. The mean concentration of NoV GII ± SD on membrane was calculated for two periods, autumn-winter (A-W) and spring-summer (S-S) for all types of membrane combined (∗p < 0.05). [file Data_Sheet_4.DOCX]

**Supplemenraty data S4**
