# Supplementary material for: Passive Samplers, a Powerful Tool to Detect Viruses and Bacteria in Marine Coastal Areas
Source: Front Microbiol. 2021 Feb 23;12:631174. doi: 10.3389/fmicb.2021.631174 (PMC7940377; doi:10.3389/fmicb.2021.631174)
Supplement: Supplementary Data Sheet 5 — Frequencies of NoV GII-positive membrane in 2017–2018. Frequencies are expressed as percentages. [file Data_Sheet_5.docx]

|  | **LDPE** | | **Nylon** | | **Zetapor** | |
| --- | --- | --- | --- | --- | --- | --- |
|  | **48 h** | **15 days** | **48 h** | **15 days** | **48 h** | **15 days** |
| **Whole 2017–2018 period** | 22.2 | 26.9 | 37.0 | 38.5 | 37.0 | 23.1 |
| **October–March** | 42.9 | 46.2 | 64.3 | 53.8 | 50 | 46.2 |
