# Supplementary material for: Passive Samplers, a Powerful Tool to Detect Viruses and Bacteria in Marine Coastal Areas
Source: Front Microbiol. 2021 Feb 23;12:631174. doi: 10.3389/fmicb.2021.631174 (PMC7940377; doi:10.3389/fmicb.2021.631174)
Supplement: Supplementary Data Sheet 6 — Recommendation for passive sampling according to microorganisms. [file Data_Sheet_6.DOCX]

|  | Membrane | Exposure time | Detection/quantification |
| --- | --- | --- | --- |
| OsHV-1 | zetapor | 48h | PCR detection |
| NoV | Nylon | 48h and 15 days | PCR detection and quantification |
| Vibrio spp | Nylon | 48h and 15 days | PCR detection and quantification |
| AllBac | Nylon | 48h and 15 days | PCR detection and quantification |
| HF183 | Nylon | 48h | PCR detection |
| Vibrio enteropathogenic | Nylon | 48h | Enrichment + PCR detection, quantification |

SD6
