# Supplementary material for: Passive Samplers, a Powerful Tool to Detect Viruses and Bacteria in Marine Coastal Areas
Source: Front Microbiol. 2021 Feb 23;12:631174. doi: 10.3389/fmicb.2021.631174 (PMC7940377; doi:10.3389/fmicb.2021.631174)
Supplement: Supplementary file 7 [file Table_1.DOCX]

|  | **Period analysed** | **All membranes** | **LDPE** | **Nylon** | **Zetapor** |
| --- | --- | --- | --- | --- | --- |
| **OsHV-1** | Spring-summer  n = 132 | 7.5 | 4.5 | 2.3 | 16 |
| ***Vibrio* spp.** | All year  n = 167 | 100 | 100 | 100 | 100 |
| ***V. alginolyticus*** | Spring-summer  n = 89 | 32.5 | 38.5 | 27 | 53.9 |
| **Sapovirus** | All year*  n = 84 | 4.7 | 0 | 6.9 | 7.4 |
| **NoV** | All year  n = 241 | 36 | 32.5 | 35 | 40.7 |
| **AllBac** | All year  n = 179 | 72 | 70 | 74.1 | 72.1 |
| **HF183** | All year  n = 134 | 13.5 | 17.8 | 13.9 | 7.14 |
