# Supplementary material for: Passive Samplers, a Powerful Tool to Detect Viruses and Bacteria in Marine Coastal Areas
Source: Front Microbiol. 2021 Feb 23;12:631174. doi: 10.3389/fmicb.2021.631174 (PMC7940377; doi:10.3389/fmicb.2021.631174)
Supplement: Supplementary file 8 [file Table_2.DOCX]

|  |  | March | April | May | June | July | August | Sept | Oct |
| --- | --- | --- | --- | --- | --- | --- | --- | --- | --- |
| **2017** | 48 h | + | + | - | + | NA | NA | NA | NA |
|  | 15 days | - | - | - | - | NA | NA | NA | NA |
| **2018** | 48 h | - | + | + | + | + | + | - | - |
|  | 15 days | - | - | - | + | - | - | + | - |
